# In Defense of Using Medical Practice Guidelines Defensively: A Response to Stewart and Peck

**DOI:** 10.1017/amj.2025.10087

**Published:** 2025-12

**Authors:** Mark A. Hall

**Affiliations:** Wake Forest University, Winston-Salem, United States

**Keywords:** Medical Malpractice, Restatement of Tort Law, Clinical Practice Guidelines, Professional Standard of Care

## Abstract

The American Law Institute (ALI) recently approved its first ever Restatement of Medical Malpractice law. One Restatement subsection embraces the position that an authoritative clinical practice guideline, if admissible and sufficiently relevant, can be prima facie evidence of a provider’s compliance with the standard of care. This article responds to a forceful critique of that position by two of the Restatement’s advisors, who are nationally esteemed members of the plaintiff’s bar. They argue that caselaw does not support this provision, that it is unsound public policy, and that the provision is unfair because it does not afford the same prima facie proof status to plaintiffs’ use of practice guidelines.

This article addresses each of those critiques. It starts with the observation that, at bottom, this opposition is fundamentally at odds with the primary governing principle of professional liability, namely, that professional standards have greater force in medical liability cases than do industry standards in general negligence cases. Because professional standards determine professional negligence, a relevant clinical practice guideline that speaks with authority for a relevant segment of medical professionals, if admissible, should be sufficient to support a jury finding of non-negligence for a doctor who complies.

The same conclusion does not apply, however, when a plaintiff presents a single relevant guideline that a physician failed to follow, for the simple reason that it is often the case that more than one approach can reasonably apply to a given clinical situation. Also, guidelines often set ideal rather than minimal standards. Thus, this provision’s differential effect is not fundamentally unfair. Instead, it flows directly from a plaintiff’s burden of establishing professional negligence, much like numerous other conventional legal rules can affect opposing sides of a case differently.

As accomplished advocates, it is no surprise these authors have made the strongest possible case against any enhanced legal status for defensive use of exculpatory practice guidelines. Thoughtful inspection and reflection reveal, though, that their analysis significantly misstates or over-states the Restatement’s position, and so their attacks are mostly misdirected. If this Restatement’s position were truly as radical or poorly considered as these authors portray, the ALI likely would not have adopted it. Nevertheless, engaging with this critique can better elucidate reasons that courts might view this Restatement provision as well-considered.

## Introduction

In *A Bridge Too Far: Practice Guidelines in the New ALI Medical Malpractice Restatement*,^1^ Larry Stewart and Robert Peck, two highly respected members of the American Law Institute (ALI), mount a forceful and sustained critique of a provision in the recently adopted Medical Malpractice Restatement^2^ that addresses defensive use of authoritative medical practice guidelines. This provision, Section 6(b), in what is expected to be the final version, states:

Evidence that the provider complied with a relevant and authoritative practice guideline may be: (1) used to rebut the plaintiff’s claim that the provider breached the standard of care … ; or (2) sufficient to find that the provider met an acceptable alternative standard of care … .^3^

Stewart and Peck argue vociferously that caselaw does not support this provision, that it is unsound public policy, and that the provision is unfair because it does not afford the same prima facie proof status to plaintiffs’ use of practice guidelines.^4^

Stewart is an emeritus member of ALI’s Governing Council and an invited adviser to the Malpractice Restatement Project, and Peck was active in that same project as part of the Members’ Consultative Group.^5^ Although both Stewart and Peck had expressed concerns about the Project’s treatment of authoritative practice guidelines — concerns that led to significant discussion and substantial revisions — no motions were made to amend or reject this portion of the Restatement (which is Section 6(b) and its Comment *f*).^6^ In 2022, ALI’s Council approved an earlier version of this provision, and in 2024 ALI’s membership approved the Project as a whole,^7^ including this provision.^8^ Both approvals occurred without significant voting dissent, and without any strong objection during the ALI’s several deliberative stages prior to final votes.^9^

It is therefore with some surprise that I read Stewart and Peck’s article advocating at some length that courts reject this provision adopted by the ALI following substantial attention.^10^ However, Stewart and Peck are nationally esteemed members of the plaintiff’s bar,^11^ and so their concerns and arguments should be taken seriously. Thoughtful inspection and reflection reveal, though, that their position is not well-founded, in large part because they misstate what this provision does and mischaracterize its support.

## Motivations: Professional versus Jury-Determined Standards

Before delving into those specifics, I step back to reflect on why Stewart and Peck appear this concerned about a provision that, by most accounts, addresses a technical issue of only modest significance. I believe their primary motivation is revealed in the article’s Section V.C., which argues that the provision would impermissibly allow physicians “to devise medical standards of care, thus affording the medical profession opportunities to enact its own standard of care to justify practices that would otherwise be deemed substandard.”^12^

Elsewhere in ALI deliberations of this Restatement, Mr. Stewart pressed the argument that juries should remain the ultimate arbiter of what constitutes reasonable medical care, rather than deferring to professional norms of competent care.^13^ Stewart and Peck’s article advances the same position — that professional standards have no greater force in medical liability cases than industry standards have in general negligence cases.^14^ They write, for instance, that the “medical profession can never set the medical standard of care,”^15^ quoting Judge Learned Hand who famously ruled in *The T.J. Hooper* that “a whole calling may have unduly lagged in the adoption of new and available devices. It never may set its own tests, however persuasive be its usages.”^16^

That foundational motivating position, however, is fundamentally at odds with the primary governing principles of professional liability. This Restatement’s Section 5, for instance, states that medical professionals are judged by the standard of care “regarded as competent *among similar medical providers* in the same or similar circumstances."^17^ Its Comment *c* “recognizes that the governing standard … ultimately, consists of what other professionals regard as competent.”^18^ In agreement, for instance, is a case the authors cite as favoring their position, but which clearly states that “[g]enerally, the standard of care for a physician is one established by the profession itself.”^19^

The ALI recently approved the same position (without dissent) in provisions addressing professional liability more broadly than just medicine.^20^ The relevant Comment *e* explains that the “reasonable professional” standard governing professional liability

displaces the ordinary rule with regard to the role of the jury in assessing breach. Generally in tort law, a jury is free to substitute its own judgment for the judgment of those within a particular business or vocation. See … The T.J. Hooper … (explaining that “a whole calling” may “never may set its own tests” as “there are precautions so imperative that even their universal disregard will not excuse their omission”).

When it comes to professionals, by contrast, “… *[i]f professionals agree that something should be done a certain way to meet the standard of care, then the jury may not second-guess that determination.*”^21^

Stewart and Peck’s motivating stance is contrary to these and several other Restatement provisions.^22^ The idea that the *T.J. Hooper* principle should apply fully to professional liability is at the root of Stewart and Peck’s pitched resistance to permitting authoritative practice guidelines serving as direct evidence of the governing standard of care. However, if courts are to follow the long-standing, thoroughly established, and widely recognized position that professional standards determine professional negligence, then Stewart and Peck are wrong. Relevant practice guidelines that speak with authority for a relevant segment of medical professionals do merit special legal attention.

## An Illustration

I now turn to what attention guidelines merit, and how that attention might sensibly differ somewhat for plaintiffs and defendants. At the outset, the following scenario illustrates the core logic of this Restatement provision:^23^

The U.S. Preventive Services Task Force, created by the Department of Health and Human Services, “is an independent group of national experts in prevention and evidence-based medicine that … [makes] evidence-based recommendations about clinical preventive services” such as screenings and preventive medications.^24^ Federal law requires health insurers to follow the task force’s leading recommendations.^25^ The appointed members are mostly primary care clinicians across a range of relevant practice areas. Members “must have no substantial conflicts of interest that could impair the integrity of the work of the Task Force.”^26^ Its recommendations “are based on a rigorous review of existing peer reviewed evidence” and, in evaluating the “effectiveness of a service,” the “Task Force does not explicitly consider costs.”^27^

One Task Force recommendation is that women with no indication of elevated breast cancer risk do not need a screening mammography more often than every two years.^28^ Suppose a physician advises a low-risk patient (after discussing the pros and cons) that annual mammographies are not necessary, but 20 months after her last screening she is found to have stage 3 breast cancer. Suppose further that the patient sues the doctor, supported by an expert witness who says that annual testing is preferred, which would have caught the cancer at an earlier, more treatable stage. In this scenario, the Restatement section provides that the doctor does not need a rebuttal witness to create a triable issue over whether the doctor complied with the standard of care.^29^ Stated another way, the defendant can survive summary judgment simply by presenting evidence of complying with this relevant, authoritative guideline.

Suppose instead that, instead of having a standard of care expert, the patient presents admissible evidence (perhaps by cross examining the defendant) that the American Cancer Society recommends annual screening mammograms. That recommendation could well be relevant evidence, but the Restatement section’s Comment *f* provides that, “unsupported by expert opinion on breach of the standard of care, the guideline is not sufficient for a factfinder to conclude that [the doctor] was negligent. Accordingly, … without more, [the patient] fail[s] to satisfy her burden of production on breach of the standard of care.”^30^

Stewart and Peck strongly object to both of these legal outcomes.^31^ They do not think the defending doctor should be able to avoid the need for a supporting expert on the standard of care by pointing to relevant practice guidelines, even if they are authoritative.^32^ And, they are especially adamant that it is unfair to give guidelines greater legal significance when used defensively than when used offensively.^33^

The straightforward logic of the Restatement’s position, however, is fairly apparent. First, a guideline that is this authoritative, relevant, and unambiguous should be sufficient to support a jury finding of non-negligence for a doctor who complies.^34^ Second, the same conclusion does not apply when a plaintiff presents a single guideline that a physician failed to follow, for the simple reason that, as the authors thoroughly document, it is often the case that more than one approach can reasonably apply to a given clinical situation.^35^ Thus, failure to take one particular approach, standing alone, is insufficient evidence to support a finding of negligence.^36^

## Hearsay

Why then the vehemence of Stewart and Peck’s opposition to this relatively uncontroversial Restatement provision? Principally, the core of their position is a mischaracterization of the provision’s legal effect. Repeatedly, they claim that the Restatement “would change the hearsay rules of evidence, since the new rule would authorize what is undeniably hearsay to be used as substantive non-hearsay evidence.”^37^ This extrapolation is not justified. As the Reporters’ Note underscores, “this Section conveys substantive legal principles rather than rules of evidence.”^38^ Thus, the provision says nothing about whether or how practice guidelines can be introduced or who qualifies as an expert; instead, it speaks only to whether, if introduced by defense, practice guidelines sometimes avoid the substantive rule that usually requires expert testimony on compliance with the standard of care.

Accordingly, the section is entirely compatible with courts continuing to regard practice guidelines as hearsay. As such, the authors are correct that guidelines would need to fall under a hearsay exception, such as judicial notice or to corroborate a witness’s testimony.^39^ Nothing in the section says, or even intimates, the contrary. Nor does this section prevent or reduce plaintiffs’ ability to challenge a guideline’s accuracy and integrity. Although recognizing a hearsay exception forecloses cross-examining a guideline’s actual drafter, plaintiffs can still call their own experts to attack or minimize a guideline. Thus, defensive guidelines remain fully rebuttable.^40^

Sometimes, it will be possible to meet a standard hearsay exception without calling an authenticating expert — such as by taking judicial notice or if an opposing witness concedes the guideline’s authenticity and status.^41^ Often, however, the authors are correct that expert testimony will be needed to establish whether a guideline is admissible and meets the section’s criteria of relevance and authoritativeness.^42^ In fact, Comment *f* states this explicitly:

As is likely clear, … some expert testimony will usually be needed to: (1) establish that the guideline, in fact, satisfies the three threshold prerequisites outlined above; (2) understand how, exactly, the guideline applies to a specific case; or (3) establish whether, in fact, the provider complied with, or deviated from, the guideline.^43^

For these reasons, the section does not convert hearsay into non-hearsay. Rather, it provides only that, in some circumstances, when practice guidelines are admissible, they can be prima facie evidence of compliance with the standard of care.

## Mischaracterizations and Exaggerations

The Restatement states simply that a practice guideline that satisfies the section’s prerequisites, “*while not conclusive, may be sufficient* to support a finding that the defendant-provider did not breach [the] standard of care.”^44^ The emphasized language just quoted points to another element of misdirection the article employs by repeatedly characterizing this section as having much stronger legal effect than this. For instance, the authors state incorrectly that this section creates a defensive “safe harbor”^45^ or “shield,”^46^ and they suggest that the section contemplates practice guidelines “conclus[ively]” “establishing” the standard of care.^47^ Explicitly to the contrary, however, Comment *f* states that this section “does not declare that guidelines are conclusive.”^48^ Nor, as the authors seem to claim, does the Restatement “believe any guideline is *more* authoritative than expert testimony.”^49^

Similarly unconvincing is the authors’ claim that the section will permit inappropriate (“through the back door”) resurrection of a now-rejected locality focus in the standard of care by encouraging the adoption of guidelines that give greater recognition to the diversity of practice norms in varying circumstances across the country.^50^ Here too, the authors’ claim finds no basis in the Restatement’s actual provisions, and its reasoning does not stand up to scrutiny. The authors harken to a time several generations ago when strictly local standards defined medical negligence.^51^ They correctly note that courts have overwhelmingly rejected that strict-locality position, but the authors fail to note that locality characteristics remain potentially highly pertinent in determining the relevant circumstances of a particular case.^52^

If relevant circumstances vary by location, then guidelines that account for those circumstances are justified. If locally focused guidelines are not based on relevant factors, then they are not sufficiently relevant to a case to meet the section’s criteria for enhanced legal recognition.^53^

Exaggerated versions of the Restatement’s position, bordering on hyperbolic, are sprinkled throughout the article. The authors state, for instance, that this Restatement provision is a “controversial and problematic experiment”^54^ that “would allow the introduction of potentially dispositive evidence without any opportunity for cross-examination.”^55^ They suggest that defensive clinical guidelines become “irrebuttable,”^56^ thus “chang[ing] the character of documents based on which party offers them, making the rule one-sided, limiting cross-examination, and tilting the scales of justice,”^57^ which is “an astonishing proposition that is inconsistent with fundamental trial fairness”^58^ and “would unquestionably be major innovations that would embark into uncharted jurisprudence.”^59^

By overstating the section’s legal significance in these ways,^60^ Stewart and Peck set up two lines of argument that appear much more formidable than is justified. First, they argue that caselaw does not support, and perhaps outright rejects, the substantive effect they claim (incorrectly) the Restatement gives to authoritative defensive guidelines.^61^ Second, they reason that the section’s differential treatment of plaintiffs and defense is grossly unfair.^62^ They develop these arguments in a classic “straw man” fashion, by attacking a distorted, wrongly imagined version of the rule that the Restatement does not actually embrace.

## Misdirected Arguments: Caselaw

While acknowledging that this section “is based more on general principles than on extensive case law,” the Reporters’ Note does cite and quote credible caselaw support.^63^ In *Arpin v. United States*,^64^ Judge Posner (writing for the 7^th^ Circuit applying Illinois law) held that a Medicare payment rule set a rebuttable standard of care with which the defendant complied.^65^ The issue arose in the context of whether a “preceptor” training physician failed to supervise a resident physician adequately.^66^ The District Court found “that it is the duty of a resident physician’s preceptor … personally to examine a patient who has already been examined by the resident … .”^67^ The core reason the Court gave for rejecting this ruling was that the trial judge “based this finding entirely on testimony by the plaintiff’s expert witness,” whose opinion was contradicted by “Medicare reimbursement rules … that excuse an attending physician from routinely having to examine or otherwise observe a resident’s patient.”^68^

Because the *Arpin* Court ultimately sustained the trial judge’s finding of negligence on a different basis, Stewart and Peck argue that this full paragraph analysis is only “a passing comment,” and that it speaks only to an expert witness’s qualifications without directly supporting the Restatement’s position.^69^ Careful reading shows, however, that this part of Judge Posner’s opinion embodies the Restatement’s position by holding as a matter of law that, where an authoritative practice guideline established a relevant standard of care, it became conclusive when not effectively rebutted by the plaintiff.^70^

The other cases the Reporters’ Note cite are not as directly on point but nevertheless endorse in some manner heightened significance for compliance with an authoritative practice guideline.^71^ The Reporters, however, were careful not to overstate the significance of this supportive caselaw. The same cannot be said for the authors’ analysis of opposing caselaw, considering that much of it is not nearly as oppositional as the authors portray.^72^

For instance, *Adams v. Laboratory Corporation of America*,^73^ which the authors cite several times,^74^ rejects exculpatory use of a particular set of guidelines but does not reject defensive guidelines as a general matter.^75^ Instead, the court concluded the guidelines in question were especially untrustworthy because they “moved away from disinterested scientific inquiry and into litigation policy to serve their members’ own interests.”^76^ Similarly *Jewett v. Our Lady of Mercy Hosp. of Mariemont* ^77^ held only that a defensive guideline was not conclusive and so did not warrant summary judgment for the physicians, considering the rebuttal evidence from the plaintiff’s expert. That holding is also entirely consistent with this Restatement section. *Yi v. New York State Bd. for Pro. Med. Conduct*,^78^ which the authors cite,^79^ addressed inculpatory use of guidelines (against a physician in a medical licensure case) rather than defensive use, and it found that the particular guidelines “do not purport to define an authoritative standard of care.”^80^ Even then, the court took judicial notice of the guidelines and gave them a great deal of credence in ruling against the physicians.^81^

## Misdirected Arguments: Differential Effects

Turning then to the section’s differential impact on plaintiffs and defense, the authors employ a favorite rhetorical flourish used by ALI members when opposing a Restatement provision they disfavor, by claiming that it will “tilt the scales of justice.”^82^ This attack also misstates or overstates the Restatement’s position. The authors claim, for instance, that “the Restatement does not cite a single case to support the limitation … that would *prevent* plaintiffs from also using practice guidelines in an inculpatory manner,”^83^ and they suggest that, under the Restatement, “the use of practice guidelines as standard of care evidence [is] *restricted in all cases to just defendant* providers” and “that plaintiffs [are] *banned* from also using [guidelines] in an inculpatory manner.”^84^

As I have already emphasized, however, the Restatement does not in any way restrict plaintiff’s use of practice guidelines, nor does it elevate the status of exculpatory guidelines to a “safe harbor” that conclusively establishes the standard of care, even when applicable guidelines are “authoritative.” Instead, the Restatement merely states that such guidelines can provide prima facie evidence of compliance with the standard of care.^85^

Nevertheless, Stewart and Peck level much of their attack on positions the Restatement does not embrace. They recount history from several decades past when “litigation critics and special interest groups sought to convert [practice guidelines] into defensive tools that would provide safe harbors for medical defendants.”^86^ More troubling, a few states that experimented with legislation creating a type of malpractice immunity for complying with official practice guidelines barred plaintiffs from presenting evidence of noncompliance.^87^ As the Reporters’ Note observes, it is this much more extreme version of bifurcated legal effect — barred from plaintiff’s evidence vs. irrefutably conclusive for defense — that commentators have roundly criticized.^88^

Even though this intellectual history is almost entirely off point, the authors cite and quote this history as if it were principally on point.^89^ What would be much more relevant, however, is whether it is fundamentally unjust to give similar evidence modestly different substantive status when presented by plaintiffs versus defendants.

Law frequently, and uncontroversially, gives differential advantage to one party over the other, in the form of burdens of proof, burdens of production, burdens of persuasion, logical or legal presumptions that require rebuttal, and the like. How fair such rules are depends (at least in part) on their justification for differential impact. Here, the justification is simply that plaintiffs bear the burden of proof, and that finding professional negligence requires showing that the defendant failed to comply with any acceptable standard of care.^90^ By simple logic, it follows that proof of compliance with just one acceptable standard of care has more legal significance than proof of noncompliance with a single standard.

This is well illustrated by litigation over FDA drug “labeling” or “package insert” standards. If a doctor complies with the FDA’s approved instructions and indications for how and when a drug should be used, that obviously constitutes prima facie (albeit not conclusive) proof of non-negligence.^91^ If a plaintiff, however, simply shows that a physician deviated from the FDA-approved instructions and indications, although that information can support expert testimony of negligence, it does not by itself establish a prima facie case.^92^ The reason is that FDA labeling does not foreclose alternative standards of care reflected in physicians’ reasonable decisions to prescribe drugs “off-label.”^93^

The same logic applies to compliance versus deviation from an authoritative practice guideline. Denying prima facie status to drug label noncompliance is not thought to be fundamentally unfair to plaintiffs,^94^ nor should the same be true for practice guidelines. Thus, expert testimony is needed to determine whether off-label deviation is reasonable.

## Public Policy Considerations: The Quality of Guidelines

Having addressed the authors’ primary legal arguments, I now turn to briefly consider their public policy arguments. Principally, they describe many ways in which guidelines can be deficient.^95^ However, this kind of argument is a classic example of the “nirvana fallacy,” rejecting a proposal because of its flaws without considering the imperfections of its alternatives.^96^

Here, the alternative to authoritative practice guidelines is individualized expert testimony. The same claims the authors make about guideline deficiencies can also plausibly be made about hand-picked adversarial experts. Consider, for instance, these passages from their article, edited to replace their target (guidelines) with the alternative (experts):

In the present-day proliferation of [expert witnesses], there are many objectivity and credibility issues, resulting in severe criticism of [expert testimony] within the profession. Many [experts] offer inconsistent and conflicting recommendations, contain inherent conflicts of interest and mixed purposes, and some are even blatant attempts to tilt the playing field in favor of defendants … .^97^

Different [experts] addressing the same topic provide inconsistent and conflicting blueprints for diagnosis and treatment because there is no “centralized authority to coordinate, vet, approve, and catalog [expert opinions],” and there is “an absence of a universal methodology to create [expert testimony].” Each [expert] that chooses to promulgate [an opinion] “decides freely which, if any, framework they will use to construct [their opinion],” and the [opinions] produced remain “susceptibl[e] to bias and conflicts of interest,” while they also often “suffer from a lack of rigor and applicability.”^98^

To bring more sharply into focus the plausibility that practice guidelines should sometimes be allowed to stand in for expert testimony, it helps to understand what it is that standard of care experts testify to. “The standard of reasonable medical care is the care, skill, and knowledge regarded as competent among similar medical providers in the same or similar circumstances.”^99^ In other words, experts address what other professionals would think about the situation. But how do experts know this? Often, they have little more to go on than personal experience.^100^ Of significance, the *Daubert* standard for expert opinion admissibility^101^ does not generally require anything more for testimony on the medical standard of care.^102^

From a perspective of methodological rigor, relevant authoritative practice guidelines can be not just *equivalent* to expert testimony, they can be a far superior way to document “what other professionals regard as competent,”^103^ because authoritative guidelines are developed through a process that directly reflects actual consensus within a relevant portion of the profession.^104^ Guideline development is based on direct engagement with a range of relevant practitioners.^105^ More than this, guidelines are also based on available scientific evidence about documented treatment outcomes.^106^ As one judge explained:

[Practice guidelines] consist of systematically developed statements designed to assist the practitioner and patient in making decisions about appropriate health care in specific clinical circumstances. Rather than being a mere sampling of professional opinion, these guidelines provide consensus standards of conduct that are both clearer and more rational than those currently used to identify professional negligence.^107^

Naturally, many guidelines do not live up to best practice standards.^108^ Nevertheless, the authors overstate the extent to which medical professionals themselves disdain practice guidelines. For instance, they point to the federal government’s closure of the National Guideline Clearinghouse as an indication that the agency in charge thought that cataloguing guidelines “no longer made sense,”^109^ but in truth this closure happened due to budget cuts during the first Trump administration.^110^ Since then the agency “has conducted various activities aimed at restoring what had been a highly valued resource of evidence-based clinical practice guidelines.”^111^

Similarly misleading is the authors’ claim that “up to fifty percent of guidelines are considered untrustworthy within the profession.”^112^ The cited support, however, is an article from the orthopedic surgery department in Barcelona Spain that makes clear this fifty percent guesstimate is based on these and other authors’ judgment of what they regard as trustworthy guidelines rather than reflecting actual views held by practicing physicians (either in the United States or in Spain).^113^

Much more could be said in defense of practice guidelines, or in response to these authors’ critique. It suffices, however, to note that Stewart and Peck’s fairly one-sided dive into this extensive literature reveals the great extent to which the research and professional communities have taken seriously the need to uphold and improve standards by which medical practice guidelines are developed and implemented.^114^

## Public Policy Considerations: The Utility of Guidelines

The Reporters’ Note observes that one reason medical literature has given practice guidelines this much attention is the “increasing recognition of a need for greater standardization and rationalization in many areas of medical practice.”^115^ In an era of rapidly increasing science skepticism, this collective professional deliberation based on credible empirical evidence takes on even more heightened importance. In this light, it is notable that the authors’ critique of guideline trustworthiness resonates in part with the anti-science sentiment currently emerging from some senior federal administrators.^116^ Reputable guidance about preferred or discredited medical approaches could be especially useful in crisis situations that are sufficiently unprecedented to lack any established professional standard to guide either practice decisions or liability determinations.

Finally, the Reporters’ Note observes that defensive use of practice guidelines could help counter the well-documented phenomenon of “defensive medicine,” by which physicians engage in excessive medical practices geared more to avoiding liability than to doing what is best for patients.^117^ As such, practice guidelines have the “potential to interrupt or reverse the cyclonic tendency that a custom-based approach engenders to continuously elevate the intensity of care beyond what is objectively reasonable.”^118^ The Reporters explain that, to counteract this problem

professional societies have issued guidelines that recommend clinical restraint when they feel that legal pressures cause excessive treatment. See Gallardo v. United States, 752 F.3d 865, 873 (10th Cir. 2014) (applying Colorado law) (discussing such guidelines); American Board of Internal Medicine Foundation, Choosing Wisely, available at www.choosingwisely.org (referencing many such guidelines designed to counteract overuse of various costly medical procedures). But, for prudential guidelines to alleviate the defensive-liability motivation to adopt excessive medical practices, medical professionals need confidence that courts will recognize the guidelines. See, e.g., Daniel Merenstein, *Winners and Losers*, 291 JAMA 15 (2004) (describing a trial where the jury rejected multiple national guidelines recommending cautious use of a blood screening “PSA” test for prostate cancer) … .^119^

## Conclusion

As accomplished advocates, it is no surprise that Stewart and Peck have made the strongest possible case against any enhanced legal status for defensive use of exculpatory practice guidelines. If the Restatement’s position were truly as radical or poorly considered as the authors portray, however, the ALI likely would not have adopted it.^120^ Closer inspection reveals that Stewart and Peck’s analysis significantly misstates or over-states the Restatement’s position, and so their attacks are mostly misdirected. Nevertheless, engaging with their argument can better elucidate reasons that courts might view the Restatement as well-considered, even if its position is one on which reasonable minds can differ.

## Footnotes

1
*See generally* Larry S. Stewart & Robert S. Peck, *A Bridge Too Far: Practice Guidelines in the New ALI Medical Malpractice Restatement*, 51 Am. J.L. & Med. 1 (2025) (Stewart & Peck’s critique of the Restatement) [hereinafter *A Bridge Too Far*].

2 Restatement (Third) of Torts: Medical Malpractice § 6(b) (A.L.I., Tentative Draft No. 2, 2024) [hereinafter Tentative Draft No. 2]. Note that the final version of the Restatement has yet to be released as of the publication of this article.

3
*Id.* This final version currently is contained in a revision document on file with the author that was sent to ALI Council members and Project advisors in June 2024.

4
*A Bridge Too Far*, *supra* note 1, at 1.

5
*Torts: Medical Malpractice*, *Participants*, A.L.I., https://www.ali.org/project/torts-medical-malpractice?active-tab=tab_2 [https://perma.cc/3993-6MPK] (last visited Sep. 27, 2025).

6 Concerns these authors raised in written comments to the Reporters and the ALI are included in their article*. A Bridge Too Far*, *supra* note 1. The transcript of the ALI’s 2024 annual meeting, on file with the author, records the Institute’s discussion of this topic and any motions that were made relating to this Restatement as a whole. *See generally* A.L.I., Transcript of Annual Meeting MM (May 20, 2024) (unpublished transcript) (on file with author) (containing transcript of Stewart and Peck raising concerns).

7 A.L.I., *Torts: Medical Malpractice Is Approved* (May 17, 2024), https://www.ali.org/news/articles/alis-torts-medical-malpractice-approved [https://perma.cc/2VJN-HY6J].

8
*Id.* However, this approval was subject to final revisions consistent with discussions preceding the approval vote. *Id.* Subsequently, the Reporters conferred with ALI’s leadership and select Council members regarding final revisions, focusing in particular on this subsection. *See* E-mail from Mark Hall, Co-Rep., Restatement Third of Torts: Med. Malpractice, to Eleanor Barrett, Deputy Dir., A.L.I., & Diane Wood, Dir., A.L.I. (July 17, 2024, at 08:15 ET) (on file with author).

9 Preceding the final vote, this Restatement, as is typical, went through several revisions that were discussed both by the ALI’s Council (which, including emeritus members, numbers over 100), and by several dozen member advisers to the project with particular expertise in its topics. *See, e.g.*, A.L.I., *supra* note 7. For discussion of ALI’s extensive deliberative process, see Catherine Biondo, *Secondary Sources: ALRs, Encyclopedias, Law Reviews, Restatements, & Treatises*, Harv. L. Sch. Libr. (Aug. 15, 2025), https://guides.library.harvard.edu/law/secondary [https://perma.cc/BV6Z-JAGX]; *How ALI Works*, ALI Advisor, https://www.thealiadviser.org/how-ali-works/ [https://perma.cc/H2AG-7QZA] (last visited Sep. 30, 2025); *Frequently Asked Questions*, A.L.I., https://www.ali.org/faq [https://perma.cc/4BE7-PM48] (last visited Sep. 30, 2025).

10
*A Bridge Too Far*, *supra* note 1, at 2. Stewart also published a shorter version of this argument with the leading plaintiff’s bar journal, the American Association for Justice (AAJ), which is the most influential plaintiff’s advocacy group and which Stewart formerly led. Larry S. Stewart, *The New Medical Malpractice Restatement*, 61 Trial 44, 44-50 (2025). That article prompted another AAJ leader to call for “forceful resistance from the plaintiff’s trial bar” to this Restatement provision. Maria Diamond, *The New Medical Malpractice Restatement: A Must-Read for Trial Attorneys Handling Medical Negligence Cases*, Trial News (May 2025), https://www.trialnewsonline.org/trialnews/library/item/may_2025/4270509/ [https://perma.cc/MX4X-ZAY2].

11
*See Larry S. Stewart*, Inner Circle Advocs., https://www.innercircle.org/LawyerSearch/Larry-Stewart [https://perma.cc/YN2F-5W77] (last visited Sep. 22, 2025) (outlining key accomplishments in Larry Stewart’s legal career); *Biographical Sketch: Robert S. Peck, Esq.*, Hist. Soc’y D.C. Cir., https://dcchs.org/sb_pdf/biographical-sketch-robert-peck/ [https://perma.cc/AW9E-DNDM] (last visited Sep. 22, 2025) (providing a resume and biographical information of Robert Peck).

12
*A Bridge Too Far*, *supra* note 1, at 22.

13
*See* Letter from Larry S. Stewart to Mark Hall, Nora Engstrom & Mike Green (Sep. 23, 2022) (on file with author); Letter from Larry. S. Stewart to Mark Hall, Nora Engstrom & Mike Green (Jan. 4, 2023) (on file with author).

14
*A Bridge Too Far*, *supra* note 1, at 14, 22.

15
*Id.* at 22.

16 T.J. Hooper, 60 F.2d 737, 740 (2d Cir. 1932).

17 Tentative Draft No. 2, *supra* note 2, § 5 (emphasis added).

18
*Id.* § 5 cmt. c.

19
*In re* Yi v. N.Y. State Bd. for Pro. Med. Conduct, 210 N.Y.S.3d 790, 794 (N.Y. App. Div. 2024) (quoting Spensieri v. Lasky, 723 N.E.2d 544, 548 (N.Y. 1999)), *aff’d* No. 50, 2025 WL 1465647 (N.Y. May 22, 2025). See the Reporters’ Note to § 5 cmt. b for additional supporting authority that is equally explicit. Tentative Draft No. 2, *supra* note 2, § 5 cmt. b. Of note, the quoted case also states that, although “clinical practice guidelines do not define a standard of care as to medical negligence,” they “are utilized to inform a physician’s decision-making process,” and the court took judicial notice of clinical guidelines relevant to the case. 210 N.Y.S.3d at 794.

20 Tentative Draft No. 2, *supra* note 2.

21
*Id.* § 101 cmt. e (alteration in original) (emphasis added).

22
*E.g.*, Restatement (Third) of the Law Governing Lawyers § 52 cmt. b (A.L.I. 2000) (explaining that the standard of care that governs lawyers, like the standard that governs other professionals, is professionally based); Restatement (Third) Torts: Liability for Economic Harm § 4 cmt. c (A.L.I. 2020) (explaining that professional standards are “an appropriate benchmark for judgment because a professional’s methods, nearly by definition, will be difficult for jurors to evaluate from scratch”).

23 This scenario is adapted from illustrations 4 and 5 in the comments to this Restatement section. Tentative Draft No. 2, *supra* note 2, § 6 cmt. f, illus. 4 & 5.

24 Agency for Healthcare Rsch. & Quality, The Guide to Clinical Preventive Services 107 (2014) (alteration in original).

25 Coverage of Preventive Health Services, 29 C.F.R. § 2590.715-2713 (2025).

26 Agency for Healthcare Rsch. & Quality, *supra* note 24, at 107

27
*Id.* at 107-08 (alteration in original).

28 U.S. Preventive Servs. Task Force, *Screening for Breast Cancer: US Preventive Services Task Force Recommendation Statement*, 331 [J]AMA 1918, 1918-30 (2024).

29 James N. Guirl, II, *The New Restatement of Torts is Revamping Medical Malpractice Law*, Guirl Law Firm (July 8, 2025), https://www.guirlfirm.com/blog/the-new-restatement-of-torts-is-revamping-medical-malpractice-law/ [https://perma.cc/W8KR-PKQZ].

30 Tentative Draft No. 2, *supra* note 2, § 6 cmt. f, illus. 5 (alteration in original).

31
*See A Bridge Too Far*, *supra* note 1, at 10-12.

32
*See id.* at 12.

33
*See id.* at 19-21.

34 Tentative Draft No. 2, *supra* note 2, § 6 cmt. f. [A]llowing the defendant-provider to rely on a relevant practice guideline established by an authoritative body is appropriate for three reasons. First, considering the results of an authoritative body’s deliberations avoids the subjective elements that often characterize an individual expert’s judgments about competent medical practice. Second, authoritative bodies generally can be expected to reflect collective professional opinion more reliably than experts hand-picked by a party’s lawyer.
*Id.*

35
*Id.* § 6 reps.’ n. cmt. f. (explaining that plaintiffs must address “whether acceptable alternative approaches exist”).

36
*See Id.*
The fact that a provider did not furnish the patient “optimal” care does not necessarily mean that the provider breached the standard of care … . [F]or many medical situations, respectable views differ … so a practice guideline may capture only one of several acceptable approaches.
*Id.*

37
*A Bridge Too Far, supra* note 1, at 16. Stewart and Peck contend that § 6(b) denies equal justice, arguing that “when practice guidelines are relied upon by defendants, they would *ipso facto* be transformed into non-hearsay, prima facie evidence.” *Id.* at 21.

38 Tentative Draft No. 2, *supra* note 2, § 6 reps.’ n. cmt. f.

39
*A Bridge Too Far, supra* note 1, at 2, 18.

40
*See infra* notes 44-49 (discussing this issue further). The authors also wrongly conflate the Section’s special requirement of guideline “authoritativeness” with the standard evidentiary requirement to establish “authenticity.” *See id.*

41 Fed. R. Evid. 803(18)(B); *A Bridge Too Far*, *supra* note 1, at 2, 18.

42
*A Bridge Too Far*, *supra* note 1, at 17; *see also* Fed. R. Evid. 402.

43 Tentative Draft No. 2, *supra* note 2, § 6 cmt. f.

44
*Id.* (alteration in original) (emphasis added).

45
*A Bridge Too Far*, *supra* note 1, at 25.

46
*Id.* at 1, 2, 15.

47
*Id.* at 13, 20 (alteration in original).

48 Tentative Draft No. 2, *supra* note 2, § 6 cmt. f.

49
*A Bridge Too Far*, *supra* note 1, at 1. In related phrasing, the authors suggest that, under the Restatement, “practice guidelines constitute the standard of care that can, standing alone, *compel* the rejection of a plaintiff’s evidence or case.” *Id.* at 14 (emphasis added).

50
*Id.* at 5.

51
*See id.* at 4-5 (describing how many states once relied on the locality standard and how one court criticized the rule as a “relic of the nineteenth century”).

52
*Id.* at 5; Tentative Draft No. 2, *supra* note 2, § 5(b). Further, the Restatement notes that “courts use locality as a proxy for other, more specific factors identified [in the black letter], such as local medical resources, the treatment options available, or alternative schools of thought.” Tentative Draft No. 2, *supra* note 2, § 5 cmt. m.

53
*See* Tentative Draft No. 2, *supra* note 2, § 5 cmt. c. (discussing how the standard of care in a medical negligence claim is based on national standards with some variation in the standard for relevant factors).

54
*A Bridge Too Far*, *supra* note 1, at 12.

55
*Id.* at 21.

56
*See id.* at 14, 19.

57
*Id.* at 24-25 (alteration in original).

58
*Id.* at 21.

59
*Id.* at 25.

60
*See supra* notes 45-51, 54-59; *see also infra* notes 83-89. Also, the authors wrongly claim that the Restatement envisions practice guidelines as a “panacea.” *A Bridge Too Far*, *supra* note 1, at 1, 3.

61
*See, e.g.*, *A Bridge Too Far*, *supra* note 1, at 16 (noting “the lack of authority for transforming practice guidelines into non-hearsay evidence” and “the absence of a single case authorizing the restriction of practice guidelines for standard of care purposes solely to defendant providers”); *id.* at 14 (“[T]he Restatement does not cite a single case to support the limitation of § 6(b) that would *prevent plaintiffs from also using* practice guidelines in an inculpatory manner.”) (emphasis added).

62
*Id.* at 21-23.

63
*See generally* Tentative Draft No. 2, *supra* note 2, § 6 reps.’ n. cmt. f (citing multiple cases throughout the Reporters’ Notes).

64 Arpin v. United States, 521 F.3d 769, 771 (7th Cir. 2008) (applying Illinois common law for medical negligence).

65
*Id.* at 773.

66
*Id.* at 772.

67
*Id.*

68
*Id.* at 772-73.

69
*A Bridge Too Far*, *supra* note 1 at 13. The theory of negligence addressed by the Medicare rule, which the appeals court rejected, is that a supervising physician must always proactively review a resident’s evaluation of a patient. *Arpin*, 521 F.3d at 773. The alternative theory that the court accepted is that the supervising physician must do an independent evaluation if the supervisor has reason to suspect that the resident’s evaluation is incorrect. *See id.* at 773-74. Holding that the latter finding is sustainable does not diminish the legal significance of the court’s initial holding that the first finding failed as a matter of law, based on the standard of care established by the Medicare rule.

70
*Arpin*, 521 F.3d at 773; Tentative Draft No. 2, *supra* note 2, § 6 reps.’ n. cmt. f. Specifically, the court stated that these particular Medicare rules “have been said to have established ‘the standard for the level of supervision that must be provided [by the attending physician] to the resident physician,’” citing to and quoting medical literature, and that the plaintiff “has failed to establish that the standard is any higher than the standard that the Medicare rules create.” *Arpin*, 521 F.3d at 773.

71
*See* Tentative Draft No. 2, *supra* note 2, § 6 reps.’ n. cmt. f (describing several cases relating to the authority of practice guidelines).

72
*See A Bridge Too Far*, *supra* note 1 at 12-14 (describing cases that have rejected the notion that “practice guidelines constitute the standard of care that can, standing alone, compel the rejection of a plaintiff’s evidence or case”).

73 Adams v. Lab’y Corp. of Am. 760 F.3d 1322, 1324 (11th Cir. 2014).

74
*A Bridge Too Far*, *supra* note 1 at 10, 14, 17, 22, 23.

75
*See Adams*, 760 F.3d at 1333-34.

76
*Id.* at 1333.

77 Jewett v. Our Lady of Mercy Hosp. of Mariemont, 612 N.E.2d 724, 726 (Ohio Ct. App. 1992).

78
*In re* Yi, 210 N.Y.S.3d at 795.

79
*A Bridge Too Far*, *supra* note 1, at 14.

80
*In re* Yi, 210 N.Y.S.3d at 795.

81
*See in re* Yi, 210 N.Y.S.3d at 794-95.

82
*A Bridge Too Far*, *supra* note 1, at 5, 12, 21, 25.

83
*Id.* at 14 (emphasis added).

84
*Id.* at 19 (alteration in original) (emphasis added); *see also id.* at 16 (emphasizing “the absence of a single case authorizing the restriction of practice guidelines for standard of care purposes solely to defendant providers”); *id.* at 25 (stating that “there is no case in any jurisdiction that has ever held that practice guidelines … should only be available to defendants”).

85
*See* Tentative Draft No. 2, *supra* note 2, § 6 cmt. f.

86
*A Bridge Too Far, supra* note 1, at 7 (alteration in original).

87
*Id.* at 10; *see also* Michelle M. Mello, *Of Swords and Shields: The Role of Clinical Practice Guidelines in Medical Malpractice Litigation*, 149 U. Pa. L. Rev. 645, 675 (2001) (highlighting the Maine Model’s “one-way street”).

88
*See* Tentative Draft No. 2, *supra* note 2, § 6 reps.’ n. cmt. f.

89
*A Bridge Too Far*, *supra* note 1, at 10.

90
*See* 4 Stuart M. Speiser, Charles F. Krause & Alfred W. Gans, American Law of Torts § 15:11 (2025).

91
*See* Thompson v. Carter, 518 So. 2d 609, 613 (Miss. 1987) (quoting Julien v. Barker, 272 P.2d 718, 724 (Idaho 1954) to emphasize that a package insert “is not conclusive evidence of standard or accepted practice in the use of the drug by physicians and surgeons … . [I]t is prima facie proof of a proper method of use, given by the maker, which must be presumed qualified to give directions for its use and warnings of any danger inherent therein.”).

92
*See, e.g.*, Salgo v. Leland Stanford Jr. Univ. Bd. of Trustees, 317 P.2d 170, 180 (Cal. Ct. App. 1957) (requiring that expert testimony accompany plaintiff’s introduction of drug instructions, based in part on the contention that “drug manufacturer’s recommendations are always conservative”); *see also* Craft v. Peebles, 893 P.2d 138, 151 (Haw. 1995) (following *Salgo*); Morlino v. Med. Ctr. of Ocean Cnty., 706 A.2d 721, 729 (N.J. 1998) (holding that violation of drug instructions without production of expert testimony is not prima facie evidence of negligence in part because “[a]fter a drug has been on the market for a sufficient period of time, . . . physicians may rely more on their own experience and the professional publications of others than on a drug manufacturer’s [instructions].” (alteration in original)).

93
*See* Mark Herrmann & Pearson Bownas, *Keep the Label out of the Case*, 103 Nw. U. L. Rev. Online 477, 480-83 (2009).

94
*See* James R. Bird, *Package Inserts for Prescription Drugs as Evidence in Medical Malpractice Suits*, 44 U. Chi. L. Rev. 398, 445-46 (1977) (noting that “the cautious bias of the FDA may make [drug package] inserts more reliable as defensive than as offensive evidence”).

95
*A Bridge Too Far*, *supra* note 1, at 24-25.

96
*See* Thomas W. Hazlett, *The Nirvana Fallacy in “Hipster Antitrust”*, 28 Geo. Mason L. Rev. 1253, 1254 (2021). Or, to paraphrase Winston Churchill’s famous aphorism, guidelines may be thought to be the worst form of evidence, until you consider all the others. *The Worst Form of Government*, Int’l Churchill Soc’y (Feb. 25, 2016), https://winstonchurchill.org/resources/quotes/the-worst-form-of-government/ [https://perma.cc/6SKR-67DH] (“[D]emocracy is the worst form of Government except for all those other forms … .”).

97
*A Bridge Too Far*, *supra* note 1, at 1 (alteration in original).

98
*Id.* at 7 (alteration in original) (citations omitted).

99
*See* Tentative Draft No. 2, *supra* note 2, § 5(a).

100
*See* Tim Cramm et al., *Ascertaining Customary Care in Malpractice Cases: Asking Those Who Know*, 37 Wake Forest L. Rev. 699, 700 (2002) (relating the physician authors’ experience that expert witnesses often base testimony about customary care either on “their own normative judgments about what practice is reasonable or appropriate, or they testify to the way they would have treated the plaintiff”); Philip G. Peters, *Empirical Evidence and Malpractice Litigation*, 37 Wake Forest L. Rev. 757, 759 (2002) (noting that expert testimony is “much more likely to describe what [testifying physicians] and their colleagues do than what most physicians actually do” (alteration in original).

101
*See generally* Daubert v. Merrell Dow Pharmaceuticals, 509 U.S. 579 (1993) (establishing the *Daubert* standard).

102
*See* Cramm et al., *supra* note 100, at 723-25 (2002); *see also* Monica Lynne Coscia, Note, *“Trust Me, I’m a Doctor:" Medical Malpractice as a Daubert-Free Zone*, 108 Geo. L.J. 1761, 1761, 1768 (2020).

103
*See* Tentative Draft No. 2, *supra* note 2, § 5 cmt. c.

104 Inst. Med., Clinical Practice Guidelines We Can Trust 4, 6-9 (Robin Graham et al. eds., 2011).

105
*Id.* at 7.

106 Ernesto Guerra-Farfan et al., *Clinical Practice Guidelines: The Good, the Bad, and the Ugly*, 54 Injury S26, S26-27 (2022).

107
*See* Frakes v. Cardiology Consultants, P.C., No. 01-A-01-9702-CV-00069, 1997 WL 536949, at *6 (Tenn. Ct. App. Aug. 29, 1997) (Koch, Jr., J., concurring).

108 Guerra-Farfan et al., *supra* note 106, at S26.

109
*A Bridge Too Far*, *supra* note 1, at 6.

110
*National Guidelines, Quality Measures Clearinghouse Shutting* Down, Ass’n Clinical Documentation Integrity Specialists (June 14, 2018), https://acdis.org/articles/news-national-guidelines-quality-measures-clearinghouse-shutting-down [https://perma.cc/9FY6-V2G5].

111
*Guidelines and Measures Updates*, Agency for Healthcare Rsch. & Quality (Oct. 2023), https://www.ahrq.gov/gam/updates/index.html [https://perma.cc/YC3V-846U].

112
*A Bridge Too Far*, *supra* note 1, at 8.

113
*See* Guerra-Farfan, et al., *supra* note 106, at S27 (stating that “depending on how their reliability is measured, up to 50% of guidelines can be considered untrustworthy”). Also noteworthy is that, in defense of a very lucrative practice doing back surgeries, which many experts believe can be done excessively, orthopedic surgeons have been notoriously hostile to practice guidelines, at one point mounting a very aggressive political campaign against the governing agency that almost succeeded in having it entirely defunded. *See* Bradford H. Gray, et al., *AHCPR and the Changing Politics of Health Services Research*, 22 Health Affs. (Web Exclusive) W3-283, W3-297-98 (2003).

114
*See* Guerra-Farfan et al., *supra* note 106, at S26 -S27 (highlighting various efforts to standardize guideline development).

115
*See* Tentative Draft No. 2, *supra* note 2, § 6 reps.’ n. cmt. f.

116
*See, e.g.*, Apoorva Mandavilli, *Kennedy Removes All C.D.C. Vaccine Panel Experts*, N.Y. Times (June 9, 2025), https://www.nytimes.com/2025/06/09/health/rfk-jr-cdc-vaccine-panel.html (noting that, in justifying removal of all CDC vaccine panel experts, Secretary Kennedy “repeated his frequent criticism that the panel ‘has been plagued with persistent conflicts of interest’”).

117
*See* Tentative Draft No. 2, *supra* note 2, § 6 reps.’ n. cmt. f; *see also* James Gibson, *Doctrinal Feedback and (Un)reasonable Care*, 94 Va. L. Rev. 1641, 1659 (2008); David Studdert et al., *Defensive Medicine Among High-Risk Specialist Physicians in a Volatile Malpractice Environment*, 293 [J]AMA 2609, 2616 (2005).

118
*See* Tentative Draft No. 2, *supra* note 2, § 6 reps.’ n. cmt. f. Commentators have noted the “feedback” loop that exists when the threat of liability induces more aggressive testing or treatment, which behavior then, under a custom-based standard, sets a new, more demanding legal standard, causing an ever-greater ratcheting up of treatment intensity. See James Gibson, *Doctrinal Feedback and (Un)reasonable Care*, 94 Va. L. Rev. 1641 (2008); James A. Henderson, Jr., *Learned Hand’s Paradox: An Essay on Custom in Negligence Law*, 105 Calif. L. Rev. 165 (2017). Discussing whether this feedback loop leads, for instance, to some unnecessary Cesarean operations, see Kayte Spector-Bagdady et al., *Stemming the Standard-of-Care Sprawl: Clinical Self-Interest and the Case of Electronic Fetal Monitoring*, 47(6) Hastings Ctr. Rep. 16 (Nov-Dec. 2017).
*Id.*

119
*Id.*

120
*See supra* note 9 (discussing the ALI’s deliberative process).

